# Antistarvation Strategies of *E. Sinensis*: Regulatory Networks under Hepatopancreas Consumption

**DOI:** 10.1155/2020/6085343

**Published:** 2020-03-22

**Authors:** Xiaoli Huang, Yang Feng, Jing Duan, Guanqing Xiong, Wei Fan, Sha Liu, Liang Zhong, Kaiyu Wang, Yi Geng, Ping Ouyang, Defang Chen, Shiyong Yang, Lizi Yin

**Affiliations:** ^1^Department of Aquaculture, College of Animal Science & Technology, Sichuan Agricultural University, Wenjiang, Sichuan 611130, China; ^2^Neijiang Academy of Agricultural Sciences, Neijiang, Sichuan 641000, China; ^3^College of Veterinary Medicine, Sichuan Agricultural University, Wenjiang, Sichuan 611130, China

## Abstract

Crustaceans have a more persistent starvation tolerance than mammals, birds, reptiles, and even fish. This study is aimed at assessing the survival strategy and regulatory mechanism of crustaceans in response to starvation through an animal model using *Eriocheir sinensis*. In the 42-day starvation experiment, the hepatopancreas was found to become the target organ, which was characterized by atrophy of the thin wall in the hepatic tubules and expansion of the lumen. During short-term starvation, *E. sinensis* activates lipid and glycogen metabolism in the hepatopancreas with lipid metabolism dominating. In lipid metabolism, there was a significant decline in triglyceride, whereas cholesterol did not change significantly. Meanwhile, the fatty acid metabolism pathway was inhibited, but autophagy increased in the hepatopancreas, which may be the selective pathway for the decomposition of intracellular substances. However, under long-term starvation, the stored energy in the hepatopancreas was depleted, and *E. sinensis* selects to consume hepatopancreatic cells and maintain energy metabolism through apoptosis, which was triggered by both the death receptor pathway and the mitochondrial pathway. In addition, cell proliferation was blocked to reduce unnecessary energy consumption.

## 1. Introduction

Starvation is the most important pressure to promote evolution and biological prosperity [1, 2]. However, different species have different tolerance to starvation. Humans are less tolerant of starvation and usually experience malignant malnutrition diseases such as stunting, wasting, and kwashiorkor during a food shortage or starvation [3]. Under normal circumstances, humans usually die if they cannot eat or drink for approximately 7 days. The length of starvation tolerance in other mammals such as *Tscherskia triton* is half a month, aquatic animals such as Cyprinidae fish can withstand starvation for 1 month [4], and the eel can withstand 3 months [5]. Interestingly, invertebrates, such as crustaceans, are generally smaller but have stronger tolerance. Shrimp and crab can often survive for up to 2 months during starvation, while *Niphargus rhenorhodanensis* and *N. virei* can live for more than a year [6]. Invertebrates have survived more glacial periods than humans and mammals, suggesting they could maintain themselves under prolonged starvation [7].

The length of tolerance that differs in animal species may be due to the different antistarvation strategies. A commonality among many organisms, which are well adapted to prolonged fasting, is that they employ strategies of metabolic rate depression during lean times. Examples are endotherms such as bats, ground squirrels, lemurs, or marmots that use hibernation [8]. Other animals, such as planarians and *Amblyrhynchus cristatus*, can adapt to starvation by shrinking in size [5, 8]. Nevertheless, crustaceans also have other strategies to tolerate and acquire a long survival. Crustaceans seem to have energy-storage organs that differ from vertebrates which could release energy quickly. Hepatopancreas is the main organ for the synthesis and secretion of digestive enzymes, the absorption and storage of nutrients such as lipids and glycogen, and play an important role in the growth and molting process. Animals like *Macrobrachium rosenbergii* even adapt to long starvation by reabsorbing organs or structures and downregulating the function of energetically costly but unnecessary organs during food scarcity [9, 10]. However, we do not yet know whether these changes are due to the animals' passive consumption in starvation or the programmed regulatory strategies they exhibit in fighting starvation. Unfortunately, few studies have reported how animals engage their antistarvation strategies and the internal regulatory mechanism.

Chinese mitten crab (*Eriocheir sinensis*) is a species of Crustacea, Decapoda, and Varunidae, which is native to the Pacific coast of China and Korea. From 1912 to 1938, *E. sinensis* invades across the European countries of the Atlantic coast [11] and spread to North America in the early 21st century [12]. As the top 100 dangerous aggressive species in the world, *E. sinensis* may resist prolonged starvation by enduring long distances in the process of biological invasion. Besides, *E. sinensis* in their native areas also suffer from prolonged starvation due to overwintering, molting, and other reasons. Therefore, this study is aimed at establishing a starvation model using *E. sinensis* to study the survival strategy of crustaceans in response to starvation and to understand the techniques of a tenacious life endowed by evolution that is forgotten in mammals.

## 2. Materials and Methods

### 2.1. Animals and Chemicals

Five hundred *E. sinensis* (weight: 8.1 ± 1.9 g, carapace width: 2.8 ± 0.27 cm, random sex, intermolt crab) were purchased from a crab farm in Liaoning, China, and acclimatized in 20 aquaria (50 L) for 1 week. One week before the formal test, the crabs were transferred to a culture box (19 cm × 12.5 cm × 7.5 cm) and raised separately to adapt to the environment. During the temporary/domestic period, the water in the tanks was pretreated with an aeration process with added multidimensional chitin (50 mg/100 L, Henan Houmu Biotechnology) to supply the minerals and trace elements required by the crab, and 20% of the culture water was renewed every day. All animal handling procedures were approved by the Animal Care and Use Committee of Sichuan Agricultural University, following the guidelines of the animal experiments of Sichuan Agricultural University, under permit number DJ-S20154603. All chemicals were of analytical grade or the highest grade available and obtained from local companies.

### 2.2. Establishment of Starvation Model

A total of 250 healthy crabs with good vitality, complete appendages, and no disability were randomly divided into two groups: the control and starvation groups. The control group was fed during the whole period, while the starvation group was not fed during the whole process. During the experiment, the water temperature was 23 ± 2°C, the pH was 7.5–8.5, and commercial feed (full-fat fish meal 15%, soybean meal 16%, peanut meal 8%, cotton meal 12%, rapeseed meal 12.2%, high-gluten flour 14%, rice bran 5%, corn germ meal 4%, corn vinasse 6%, soybean phospholipids oil 1.5%, active attapulgite clay 3%, calcium hydrogen phosphate 2%, Chinese medicine additive *Eupatorium lindleyanum* 0.15%, salt 1.0%, and chitin 0.15% [13]) (Kangda, Huian, China) for crabs was fed at 5% crab weight (1 time/day, feeding time was 6:00–7:00 in the evening). The experiment period was 42 days, and the death and activity of the crabs in each group were observed and recorded. Twelve test crabs of each group were randomly collected at 0, 7, 14, 21, 28, and 42 days, and the body weight (the surface water was absorbed by a filter paper) was weighed. Then, the breastplate of the crabs was dissected, the gross lesions were examined, the hepatopancreas was weighed, and the hepatopancreas index was calculated (hepatopancreas index = weight of hepatopancreas/weight of the crab × 100%).

### 2.3. Light Microscopy

Four crabs in each group were necropsied at 7 and 42 days. The hepatopancreas was fixed in AFA Davidson's fixative and routinely processed in paraffin. The hepatopancreas was also trimmed into cassettes, dehydrated in graded ethanol solutions, cleared in xylene, and embedded in paraffin wax. Sections (5 *μ*m) were prepared for hematoxylin-eosin (H&E) (Besso Biotechnology, Zhuhai, China), Periodic Acid-Schiff (PAS) (Solarbio, Beijing, China), and TdT-mediated DUTP nick end labeling (TUNEL) (Roche, Basel, Switzerland) staining for microscopic analysis.

Four crabs were randomly selected from each group. The hepatopancreas was dissected and fixed in AFA Davidson's fixative for 24 h. The sample was cut into small pieces of about 0.5 cm × 0.5 cm × 0.5 cm, placed on tissue support, and frozen. After the embedding agent was placed in a cryostat, sections (5 *μ*m) were stained with Oil red O (Sigma-Aldrich, Beijing, China) at a low temperature for microscopic analysis. The relative optical intensity (ROD) of Oil red O and PAS was detected in Image Processing and Analysis in Java (Image J) 1.6.0 (National Institutes of Health, USA).

### 2.4. Electron Microscopy

Four crabs in each tank were sampled at 7 and 42 days for an ultrastructural examination. The hepatopancreas was rapidly fixed with 4% glutaraldehyde and postfixed in 2% veronal-acetate buffered OsO_4_. After dehydration in graded alcohol, the tissues were embedded in Araldite. The blocks were sectioned in a microtome with a glass knife. Sections, 65 nm thick, were placed in uncoated copper grids. The sections were stained with uranyl acetate and poststained with 0.2% lead citrate. The subcellular structure of hepatopancreas was examined with a Hitachi H-600 transmission electron microscope (TEM).

### 2.5. Lipid Detection

Four crabs were randomly selected at 7 and 42 days from each group, and the hepatopancreas was taken immediately after dissection. Then, the crabs were frozen in liquid nitrogen and stored at -80°C. Tissue weight was accurately weighed. Nine times the volume of absolute ethanol was added according to the ratio of weight (g): volume (mL) = 1 : 9. Mechanical homogenization was carried out in the ice-water bath, and then, the sample was centrifuged at 600 gravitational acceleration (*g*) for 10 minutes. The supernatant was taken for testing. The triglyceride (TG), total cholesterol (T-CHO), low-density lipoprotein cholesterol (LDL-C), and high-density lipoprotein cholesterol (HDL-C) (NJJCBIO, Nanjing, China) contents were measured according to the kit instructions.

### 2.6. Quantitative Real-Time Polymerase Chain Reaction (qPCR) Analysis

At 7 and 42 days, four crabs from each group were anesthetized by ice. The hepatopancreas was sampled, placed in an RNA/DNA protective solution (Takara, Dalian, China), and stored at 4°C. The hepatopancreas was then homogenized by crushing with a mortar and pestle and stored at -80°C. Total RNA was isolated from the hepatopancreas with an animal tissue total RNA extraction kit (Fuji, Chengdu, China). Complementary DNA (cDNA) was synthesized from 1 *μ*g of RNA using a PrimeScript RT reagent kit with gDNA Eraser (TaKaRa). qPCR was performed using an SYBR Premix Ex TaqTM Perfect Real Time kit (TaKaRa) and a Thermo Cycler (Bio-Rad, Hercules, CA, USA). *β*-Actin and *GAPDH* were used as reference genes to determine the relative expression of target genes [14]. The design of the primers used for qPCR was based on the transcriptome constructed local database (NCBI: SRR9599542), which was identified by sequencing (Table 1).

For qPCR, a 10 *μ*L reaction mixture contained 5 *μ*L SYBR Green PCR Master Mix, 3 *μ*L diethylpyrocarbonate-treated water, 0.4 *μ*L of forward primer, 0.4 *μ*L of reverse primer, 0.2 Rox II, and 1 *μ*L cDNA. The following program was used for the reactions: 3 min at 95°C for 1 cycle, samples were amplified for 40 cycles at 95°C for 10 s, melting temperature according to the specific primer pair for 30 s, followed by 10 s at 95°C and 72°C for 10 s. To distinguish between specific and nonspecific reaction products, a melting curve was obtained at the end of each run. The 2^−*ΔΔ*CT^ method was used to calculate relative changes in mRNA transcript expression from the qPCR results (ΔCT = CT_target gene_ − CT_*β*−actin_, ΔΔCT = ΔCT_experimental_ − ΔCT_control_) [15].

### 2.7. Hemolymph Glucose Measurements

Four crabs were randomly selected from each group at 7 and 42 days, and 1 mL of hemolymph was collected from the base of the walking foot. The supernatant of hemolymph was collected by centrifugation after 2 hours. The supernatant was transferred to a disposable collection bottle (IDEXX Catalyst), and then, the blood glucose concentration was measured by IDEXX Catalyst One (USA).

### 2.8. Total Protein Detection

Total protein content was detected by a modified Coomassie Brilliant Blue G-250 staining method [16]. A standard curve was created with a bovine serum albumin standard solution before the total protein content was measured. Four crabs in each group were randomly selected at 7 and 42 days, and the hepatopancreas was taken immediately after dissection. Tissue weight was accurately weighed. Then, physiological saline was added according to the ratio of weight (g): volume (mL) = 1 : 9. The sample was homogenated mechanically under ice-water conditions and centrifuged for 10 minutes at 2,500 r/min. The supernatant was diluted into 1% tissue homogenate with physiological saline at a ratio of 1 : 9. The reaction mixture was measured in a Thermo Scientific Microplate Reader Varioskan LUX (Thermo, Shanghai, China) at a wavelength of 595 nm, and the content of soluble protein was calculated according to the standard curve.

### 2.9. Flow Cytometry Assay

Four crabs were randomly selected from each group, and the hepatopancreas was dissected and placed in iced PBS (0°C). The hepatopancreas was immediately minced to form a cell suspension and filtered through a 300-mesh nylon screen. Cells were washed twice with cold PBS, and the cell pellet was resuspended at a concentration of 1 × 10^6^ cells/mL in PBS. The cell cycle was detected using the cell suspension. Then, cell apoptosis was determined by using Annexin V-FITC stain (Thermo, Shanghai, China), and mitochondrial membrane potential (MMP) depolarization was determined by JC-1 stain (Becton Dickinson, New Jersey, USA) and detection with Cyto FLEX flow cytometry. Cyt Expert was used to analyzing the data.

### 2.10. Statistical Analysis

The results are expressed as means ± standard deviations. The significance of differences was determined with analysis of variance. Each indicator was tested with one-way analysis of variance and a *t*-test. However, prevalence and mortality were analyzed with the Kaplan–Meier method and the log-rank test, respectively, to determine whether the differences between the groups were significant (SPSS v.20.0, IBM Corp., New York, NY, USA). A value of *P* < 0.05 was considered significant, and *P* < 0.01 was considered highly significant.

## 3. Results

### 3.1. Hepatopancreas Is the Main Organ Affected in *E. sinensis* under Starvation

**Figure 1:**
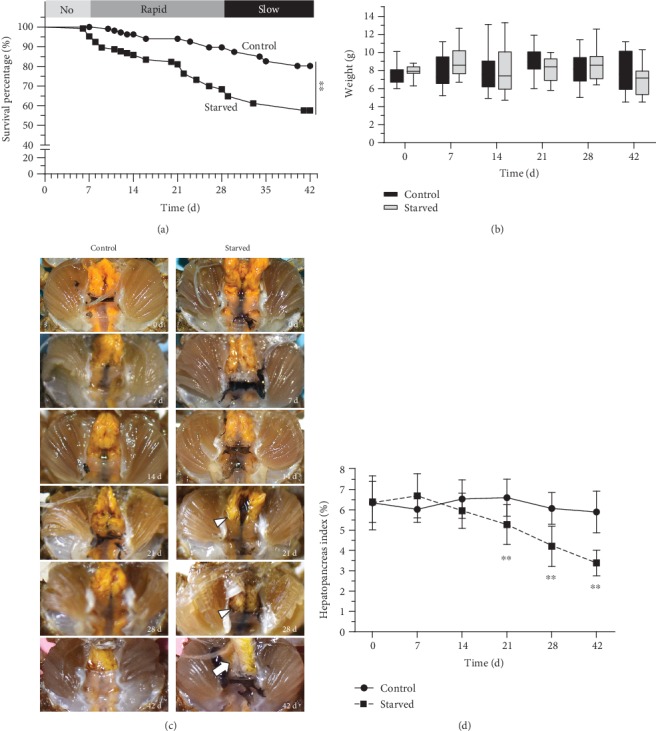
Effect of starvation on gross lesions of *E. sinensis*. (a) The survival rates of *E. sinensis* after feeding and under starvation. (b) Body weight change of *E. sinensis* under starvation. (c) The hepatopancreatic gross lesions of *E. sinensis* after starvation. ▲: hepatopancreas atrophy with hemolymph effusion; →: part of the hepatic tubule is white. (d) The hepatopancreas index of *E. sinensis* under starvation. Results are shown as mean ± SD, *n* = 12. ^∗^*P* < 0.05 and ^∗∗^*P* < 0.01 represent a significant difference and highly significant difference, respectively, between the control and the starved groups.

The present study showed that starvation could affect the survival rate of *E. sinensis*. There was no significant change in survival rates in the starved group from 0 to 7 days, but the change increased significantly after 7 days. The log-rank test showed that the survival rate of crabs in the starved group was significantly lower than that in the control group (*P* < 0.01) (Figure 1(a)), indicating that starvation had an obvious impact on *E. sinensis*. However, more than 50% of the crabs remained alive at 42 days, demonstrating that *E. sinensis* could endure long periods of starvation. According to the mortality rate of each week, the death of *E. sinensis* is presented in three stages: no death period: 0%; rapid death period: 3.2%-8.8%/week, 1.28%/d; and slow death period: 0.8%-1.6%, 0.77%/d (Figure 1(a)). This may indicate that *E. sinensis* has shown different regulatory mechanisms under prolonged starvation, thereby slowing death. Conversely, there was no significant change in the body weight of *E. sinensis* between the starved group and the control group (Figure 1(b)).

Even though the hepatopancreas was the organ mainly affected in *E. sinensis* under starvation, no gross changes were observed in the hepatopancreas during short-term starvation (0–7 days). However, there were signs of atrophy in the hepatopancreas with varying amount of hemolymph effusion in the body cavity at 14–21 days (Figure 1(c)). Moreover, when starvation lasted for 28–42 days, the hepatopancreas atrophied further, the body cavity evidently accumulated effusion, and the color of hepatic tubules became whiter (Figure 1(c)). A similar result was found in the hepatopancreas index. The hepatopancreas index of crabs in the starved group began to decrease gradually at 14 days and reached significance compared with the control group at 21, 28, and 42 days (Figure 1(d)). However, other organs did not show significant changes.

### 3.2. Hepatopancreatic Atrophy May Be a Survival Strategy for *E. sinensis* to Cope with Starvation

**Figure 2:**
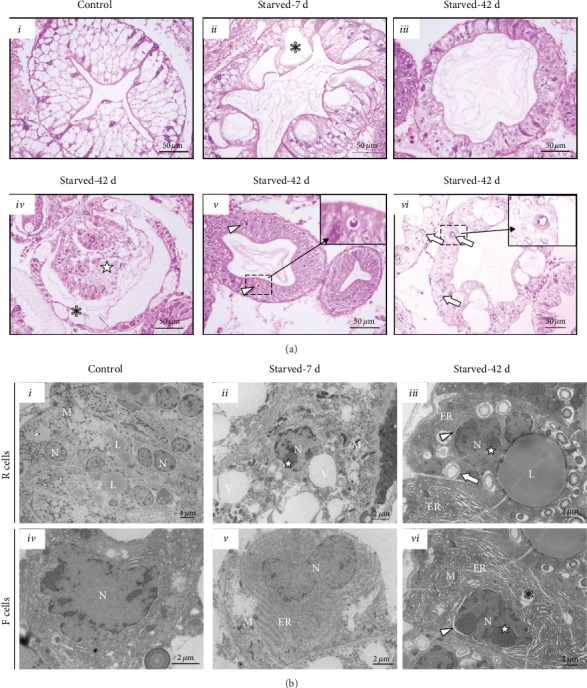
Pathological changes in the hepatopancreas of *E. sinensis* under starvation. (a) Histopathological changes of hepatopancreas after feeding (i) and under starvation (ii–vi). ^∗^B cells; arrowhead: apoptotic R cells in the tubule; arrow: nucleus swelling, cell necrosis. Scale bar: 100 *μ*m and 50 *μ*m are represented ×100 and ×400, respectively. (b) Ultrastructural pathology in the hepatopancreas of fed (i, iv) and starved (ii–iii, v–vi) *E. sinensis*. ^∗^Endoplasmic reticulum blistering. Arrowhead: nuclear membrane separation. Arrow: autophagy endosomes. ★: chromatin condensation. ER: endoplasmic reticulum. L: lipid droplet. N: nucleus. V: vacuoles.

According to the survival rate, hepatopancreas index, and gross lesions of *E. sinensis* under starvation, the effects of starvation could be divided into short-term (0–7 days) and long-term (28–42 days) stages. The normal structure of the hepatopancreas shows a stellate lumen, rich in R cells and scarce in B cells (Figure 2(a) i). At 7 days of starvation, B cells increased, R cells decreased, lumen gradually expanded, star structure disappeared, and numerous eosinophilic inclusions were found in the lumen of the hepatopancreas (Figure 2(a) ii). After 42 days of starvation, hepatic tubules still showed a great increase of B cells and a decrease of R cells, and the lumen was obviously dilated and filled with numerous content (Figure 2(a) iii). Moreover, some R cells showed apoptosis and enlargement of the nucleus, and there was necrosis in the hepatic tubules (Figure 2(a) iv–vi). There were no obvious pathological changes in the tubule interstitium and F cells of hepatic tubules under starvation.

Under TEM, the R cells in the control group contained plenty of mitochondria and numerous lipid droplets in the cytoplasm (Figure 2(b) i), and F cells contained plenty of mitochondria, endoplasmic reticulum, and ribosomes (Figure 2(b) iv). After 7 days of starvation, the nucleus of R cells shrank, chromatin edges shifted, lipid droplets decreased, and numerous vacuoles appeared in the cytoplasm (Figure 2(b) ii). Furthermore, the rough endoplasmic reticulum and ribosomes decreased in F cells (Figure 2(b) v). After 42 days of starvation, the chromatin edge of R cells migrated and became concentrated and necrotic. The microvilli of R cells became shorter, the number of lipid droplets in the cytoplasm decreased, and numerous autophagic substances appeared in the cells that showed gradually degraded substances (Figure 2(b) iii). Moreover, the rough endoplasmic reticulum and ribosomes of F cells decreased significantly, the nucleus shrank, and the chromatin edge shifted. In addition, the endoplasmic reticulum expanded and foamed in many places (Figure 2(b) vi).

**Figure 3:**
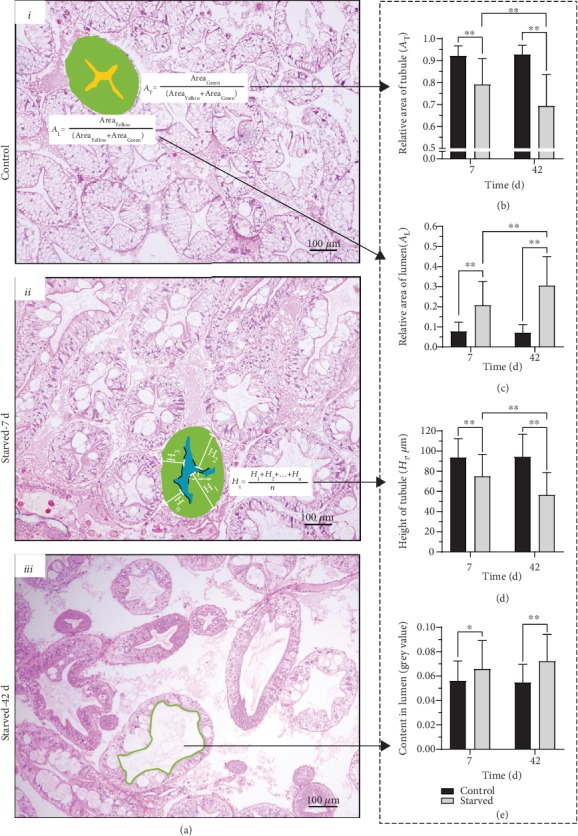
Hepatopancreatic atrophy in different experimental groups. Histology of hepatopancreatic tubule were shown in (a), as well as the relative area of tubule in (b), the relative area of lumen in (c), the height of tubule in (d), and lumen content in (e). The results were detected by ImageJ. ^∗^*P* < 0.05 and ^∗∗^*P* < 0.01 represent a significant difference and highly significant difference, respectively.

According to the pathological examination, atrophy was the main feature of the hepatopancreas under starvation (Figure 3(a) i–iii). At 7 days of the experiment, the lumen was significantly wider (Figure 3(b)), the relative area of tubule was significantly reduced (Figure 3(c)), the tubule wall became thinner (Figure 3(d)), and the contents of the lumen increased (Figure 3(e)). The atrophy of the hepatopancreas further increased at 42 days (Figures 3(b)–3(e)). Therefore, *E. sinensis* may mobilize some reactions in the hepatopancreas to cope with starvation by causing the atrophy. Based on the H&E staining and TEM observations, these reactions may be related to energy metabolism, autophagy, and cell death.

### 3.3. *E. sinensis* Consumes Hepatopancreas Energy in Short-Term Starvation to Maintain Vital Signs

#### 3.3.1. Energy Metabolism Dominated by Lipid Consumption

**Figure 4:**
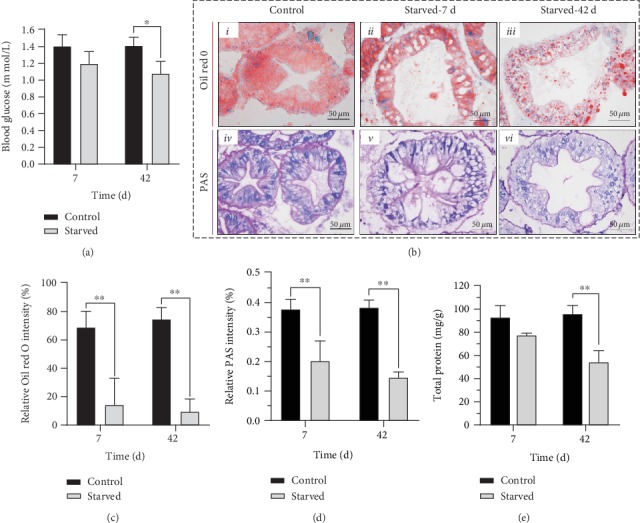
Energy metabolism in the hepatopancreas of *E. sinensis* under starvation. (a) The concentration of hemolymph glucose of *E. sinensis*. (b) Oil red O (i–iii) and PAS (iv–vi) staining in the hepatopancreas of *E. sinensis* after feeding and under starvation. (c, d). ROD of Oil red O (c) and PAS (d) in the hepatopancreas of *E. sinensis*. (e) The content of protein in the hepatopancreas of *E. sinensis*. Results are shown as means ± SD, *n* = 4. ^∗^*P* < 0.05 and ^∗∗^*P* < 0.01 represent a significant difference and highly significant difference, respectively, between the control and the starved groups.

The hepatopancreas stores substances used for energy in *E. sinensis* [17]; therefore, we detected the metabolic level of saccharides and lipid in the hepatopancreas under starvation. The results showed only a slight decrease in blood glucose after 7 days (Figure 4(a)), which may indicate that *E. sinensis* maintained basic vital signs under short-term starvation. However, Oil red O staining showed the lipid content decreased dramatically at 7 days and reached a highly significant difference between the control group and the starved group (Figures 3(b) ii and 3(c)). A similar phenomenon was observed in the PAS staining which showed that the glycogen content was severely decreased and reached a highly significant difference between the two groups at 7 days as well (Figures 4(b) and 4(d)). These results indicate that there was a significant metabolic reaction in the hepatopancreas under starvation, which according to the ROD results was based on glycogen and lipid metabolism and dominated by lipid consumption.

Although the lipid and glycogen content also reduced at 42 days and reached a highly significant difference between the two groups, the values of the two substances were not reduced ulteriorly (Figures 4(b) iii and vi, 4(c), and 4(d)). This may indicate that *E. sinensis* used lipid and glycogen as the main energy source when faced with short-term starvation. Interestingly, the total protein content in hepatopancreas did not change significantly at 7 days but was highly significantly decreased at 42 days in the starved group (Figure 3(e)).

#### 3.3.2. Triglyceride-Based Lipid Metabolism and Locked Lipid Metabolic Pathway

**Figure 5:**
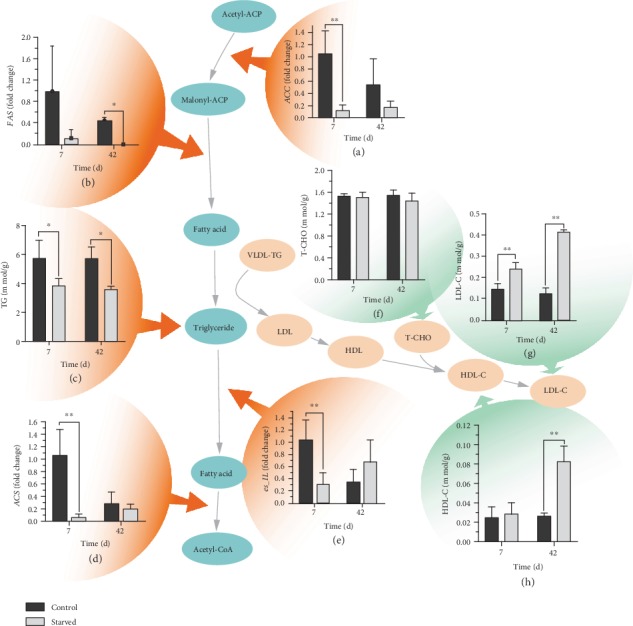
Lipid metabolism pathways of *E. sinensis* under starvation. Results are shown as means ± SD, *n* = 4. ^∗^*P* < 0.05 and ^∗∗^*P* < 0.01 represent a significant difference and highly significant difference, respectively, between the control group and the starved group.

Triglycerides are the principal form in which crustaceans store fatty acids [18]. Their synthesis and decomposition are principally regulated by acetyl-CoA carboxylase (*ACC*), fatty acid synthase (*FAS*), intracellular triacylglycerol lipase (*es_IL*), and fatty acyl-CoA synthase (*ACS*) genes [19, 20]. To reveal the mechanism of lipid consumption in different periods, we assessed the genes in the synthesis and decomposition pathway of triglycerides. Results in the present study showed that the triglyceride content decreased significantly under starvation (Figure 5(c)), while there was no significant change at 42 days compared with 7 days, indicating that triglycerides were primarily used in short-term starvation, and this was similar with the results of total lipid changes. In addition, the expression levels of *FAS* and *ACC* decreased significantly during the starvation period (Figures 5(a) and 5(b)), indicating that the synthesis of triglycerides was blocked under starvation. However, the expressions of *es_IL* and *ACS* were significantly downregulated as well (Figures 5(d) and 5(e)), and only *es_IL* showed upregulation at 42 days (Figure 5(e)), demonstrating that the fatty acid degradation pathway was also blocked.

Cholesterol, another important form of lipid, is the main component of the cell membrane [21]. The results showed that the contents of T-CHO did not change significantly under starvation (Figure 5(f)), indicating that cholesterol was not the main form of lipid used under starvation. However, cholesterol seemed to become unstable under long-term starvation. Lipoprotein is an important carrier of lipid transport and metabolism in cells and has different forms of density. High-density lipoprotein can bind with cholesterol to form lipoprotein cholesterol and promote the degradation of cholesterol [21, 22]. LDL-C increased significantly at 7 and 42 days (Figure 5(g)), and HDL-C increased significantly at 42 days (Figure 5(h)). Although low levels of HDL-C and LDL-C did not affect the overall change in cholesterol, the results indicated that cholesterol gradually became unstable as the length of starvation was prolonged.

#### 3.3.3. Autophagy May Be an Alternative Pathway for Fatty Acid Degradation

**Figure 6:**
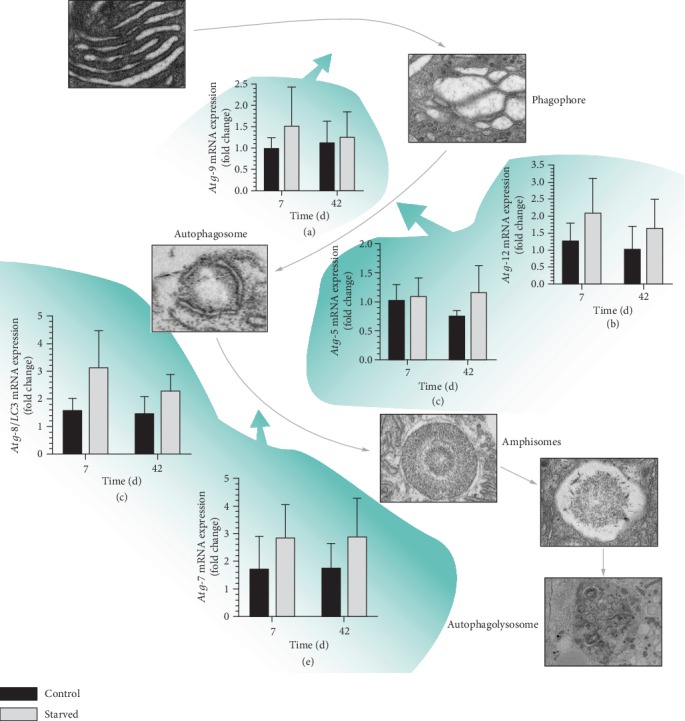
Autophagy pathways of *E. sinensis* under starvation. Results are shown as means ± SD, *n* = 4. ^∗^*P* < 0.05 and ^∗∗^*P* < 0.01 represent a significant difference and highly significant difference, respectively, between the control group and the starved group.

The fatty acid degradation pathway is a slow energy pathway that may not be suitable for starvation. Our study found that the fatty acid degradation pathway was locked. Therefore, *E. sinensis* may utilize a more efficient pathway to degrade lipids. Research has found that, under stress such as starvation, cells may degrade intracellular substances by enhancing autophagy to maintain normal growth [23]. It is commonly known that autophagy can degrade intracellular substances to maintain the balance of the cells and organs [24]. However, a previous study found that fat droplets in hepatocytes are degraded by autophagy [25]. Meanwhile, in the present study, phagophores, autophagosomes, amphisome-like substances, and autolysosomes were observed by TEM in the hepatopancreas cells of the starved group (Figure 6). Further, the autophagy-related protein (Atg) family plays an important role during the formation of autophagy [26], and we also detected the expression of Atg-related genes in hepatopancreas. The results showed the expression levels of *Atg9*, *Atg12*, *Atg5*, *Atg7*, and *LC3* were upregulated in varying degrees under starvation (Figures 6(a)–6(e)), which indicated starvation promoted autophagy of hepatopancreatic cells.

### 3.4. *E. sinensis* Consumes Hepatopancreatic Cells in Long-Term Starvation to Preserve Self-Survival

#### 3.4.1. Hepatopancreatic Cells Are Consumed under Long-Term Starvation

**Figure 7:**
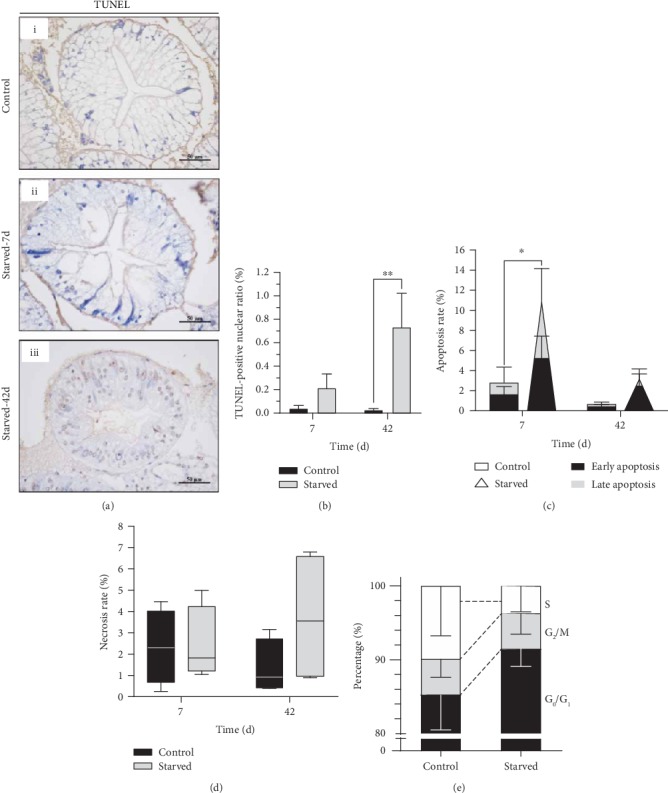
Cell death in the hepatopancreas of *E. sinensis* under starvation. (a) TUNEL staining (i–iii) in the hepatopancreas of *E. sinensis* after feeding and under starvation. (b) TUNEL-positive nuclei in the hepatopancreas of *E. sinensis*. (c) The cell apoptosis rate. (d) Cell necrosis rate of hepatopancreas. (e) The cell cycle phase compositions of hepatopancreas. Results are shown as means ± SD, *n* = 4. ^∗^*P* < 0.05 and ^∗∗^*P* < 0.01 represent a significant difference and highly significant difference, respectively, between the control group and the starved group.

Under long-term starvation, we found that blood glucose significantly decreased, which indicated that energy in the hepatopancreas has been exhausted and implied that *E. sinensis* begins to reduce self-metabolism (Figure 4(a)). It also prompted *E. sinensis* to select other strategies to resist starvation. In the present study, by using TUNEL staining and flow cytometry measurements, we found that *E. sinensis* tended to induce the apoptosis of hepatopancreatic cells under long-term starvation. In detail, after short-term starvation, a small number of apoptosis-positive signals were found in both B cells and R cells by TUNEL staining (Figure 7(a) ii, brown nuclei). In long-term starvation, the cells showed a dense apoptosis-positive signal (Figure 7(a) iii, brown nuclei), and the TUNEL-positive nucleus showed that cell death was not increased significantly at 7 days but significantly at 42 days (Figure 7(b)). Furthermore, the hepatopancreatic cells apoptotic rate increased in the starved group at 7 and 42 days, and the difference was significant at 7 days compared with the control group when detected with flow cytometry (Figure 7(c)). In addition, there was an increased rate of necrosis after 42 days of starvation (Figure 7(d)). These results indicated that *E. sinensis* activated apoptosis under starvation, which may be an alternative energy source that was used to cope with long-term starvation.

In addition, we also examined changes in cell proliferation capacity. Under starvation, the cells in the intercellular phase (G_0_/G_1_) showed an upward trend; the number of cells in the premitotic and mitotic (G_2_/M) cells did not change significantly, while the cells in the DNA synthesis phase (S) decreased (Figure 7(e)). The results indicated that *E. sinensis* stalled cell proliferation under starvation to save energy, which may be one of the causes of hepatopancreas atrophy.

#### 3.4.2. Death Receptor Pathway and Mitochondrial Pathway Are Activated to Ensure Apoptosis

**Figure 8:**
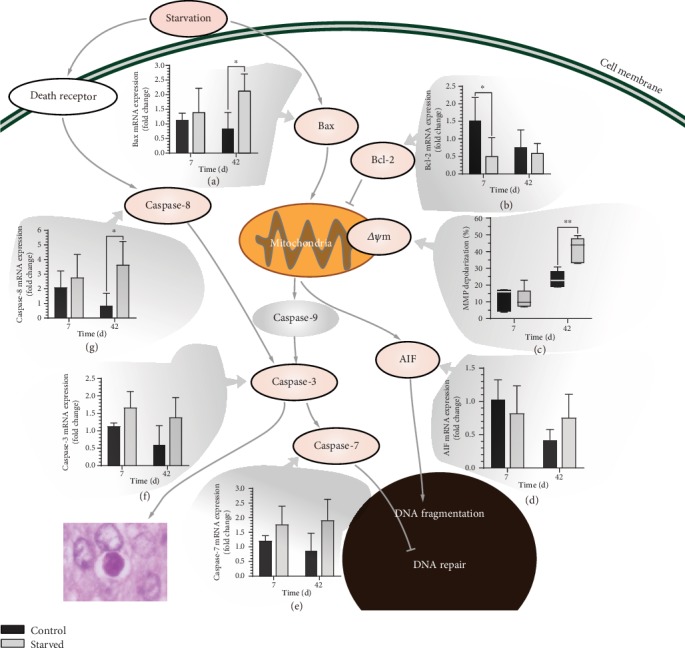
Apoptosis pathways of *E. sinensis* under starvation. Results are shown as means ± SD, *n* = 4. ^∗^*P* < 0.05 and ^∗∗^*P* < 0.01 represent a significant difference and highly significant difference, respectively, between the control group and the starved group.

Generally, apoptosis-related genes, especially terminal apoptotic effectors *caspase* (*casp)-3* and *casp-7*, in hepatopancreas cells are upregulated in varying degrees under starvation [27]. In this study, the expression of *casp-3* and *casp-7* in the starved group was upregulated at 7 and 42 days, which confirmed that starvation led to the apoptosis of *E. sinensis* hepatopancreas cells (Figures 8(e) and 8(f)).

In addition, genes expressed in the different apoptotic pathways were detected in the present study [28, 29]. First, the flow cytometry results found that the MMP depolarization rate increased significantly after 42 days starvation (Figure 8(c)). The MMP of hepatopancreas cells decreased severely, which may result in an increase of mitochondrial membrane permeability and cell apoptosis through the mitochondrial pathway [30]. Furthermore, the expression of mitochondrial pathway-related genes such as *Bax* and *AIF* was upregulated at 42 days (Figures 8(a) and 8(d)), while the expression of *Bcl-2* in the starved group was downregulated at 7 and 42 days (Figure 8(b)), indicating mitochondrial pathways were activated under long-term starvation [31].

In addition, as a marker protein of the death receptor pathway, *casp-8* was upregulated at 7 and 42 days and reached a significant difference at 42 days compared with the control group (Figure 8(g)), indicating the death receptor pathways were activated under long-term starvation [32]. In other words, *E. sinensis* may activate multiple apoptotic pathways in response to starvation and this was evident at 42 days, which could ensure the smooth progression of apoptosis and transform cells into energy.

## 4. Discussion

Crustaceans generally have a strong tolerance of starvation, and their antistarvation strategies are worthy of attention. In this study, we used *E. sinensis* as a model to reveal the strategies of crustaceans in resisting long-term starvation and to explore the related regulatory networks. Under starvation, a large cellular reaction and structural changes occurred inside the hepatopancreas, causing atrophy. These cellular responses played a key role in providing energy for *E. sinensis*. Under short-term starvation, *E. sinensis* used autophagy to ensure the lipids and carbohydrates were rapidly utilized, with lipid consumption predominant. However, under long-term starvation, the stored energy in the hepatopancreas was depleted, and *E. sinensis* further selected to consume hepatopancreatic cells and maintain energy metabolism through apoptosis. In addition, cell proliferation was blocked to reduce unnecessary energy consumption. This strategy may be an ability in crustaceans that has evolved over the centuries, such as in various glacier centuries (Figure 8).

Evolution gives animals the ability to store energy in the face of various environments. Animals such as mammals, reptiles, and fish have well-developed adipose tissue to store unused energy in normal conditions [33]. In addition, these animals also store glycogen in the liver, muscles, and other parts [34]. Under starvation, the energy in these organs will be used to supplement basic vital metabolism. However, depending on organ function differentiation, these energy-storing organs may not have developed energy degradation capabilities [33]. The adipose tissues usually degrade lipids into free fatty acids and release them into plasma under the stimulation of hormone-sensitive lipase or adipose triglyceride lipase, and they are further transported for oxidation [35]. Therefore, the imperfect energy-storage organs in crustaceans may be more advantageous in the face of starvation. Crustaceans, including *E. sinensis*, generally rely on the hepatopancreas to store lipids, which is a midgut accessory gland that combines digestion [36]. This may mean that crustaceans use energy storage more efficiently, which is especially important in conditions of extreme starvation. This organ ensures that the energy can be utilized when needed, explaining why crustaceans could endure starvation for a long time.

The way in which crustaceans resist long-term starvation may also be related to their efficient use of lipids. The fatty acid metabolic pathway is a common lipid degradation pathway [37]. However, *β*-oxidation of fatty acids suggests that their rate of degradation may not be suitable for the rapid supply of energy [38]. Autophagy could ensure the intracellular substances are rapidly metabolized. In previous studies, under stress conditions such as starvation, cells may degrade their intracellular substances by enhancing autophagy to maintain normal growth [23]. Moreover, a previous study found that fat droplets in hepatocytes are degraded by autophagy [25]. The study found that crustaceans did not activate their fatty acid metabolism pathways but chose to activate autophagy under extreme starvation, which ensured a supply of energy. In addition, autophagy-mediated fatty acid degradation also exists in eukaryotic cells such as in mice liver cells and yeast cells [25, 39]. However, this mechanism is more often used as a selective lipid degradation pathway by advanced animals, and its degradation efficiency is not understood.

Apoptosis is a common physiological state in living organisms. Studies have shown that starvation can cause apoptosis in the liver of humans [40] and rainbow trout [41], and in the hepatopancreas of *Periplaneta americana* [42], etc. However, excessive apoptosis often leads to disorders in organs and even body functions [43]. The hepatopancreas does not seem to play a major role in the physiological functions of crustaceans; for instance, in *E. sinensis*, the hepatopancreas withers during ovary development [44]. Therefore, when energy is depleted in crustaceans, the degradation of the hepatopancreas could be activated to ensure cell transformation and energy supply, which is not as easy in mammals and fish with more complete organ differentiation.

## 5. Conclusion

**Figure 9:**
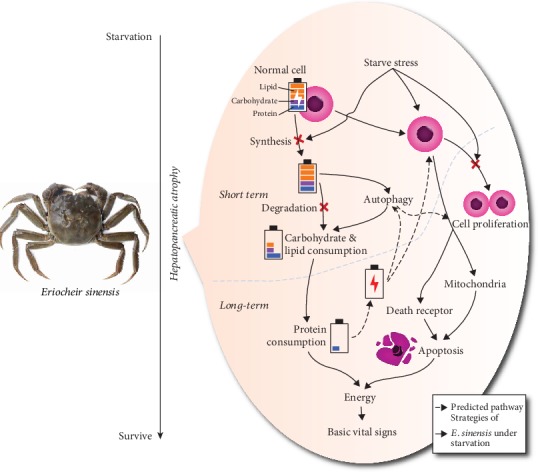
Regulatory networks of *E. sinensis* in antistarvation.

In this study, we found that *E. sinensis* had an effective survival strategy under starvation, and it could maintain survival by consuming its hepatopancreas. Through the starvation model, we found that the mortality rate of *E. sinensis* showed different stages (no death period, high death period, and slow death period). Thus, we speculated that under different degrees of starvation conditions, *E. sinensis* showed different regulatory mechanisms. The short-term starvation results in a depletion of the energy stored by the crab, whereas the long-term starvation results in a regulatory mechanism at the consumption of the hepatopancreas. Under short-term starvation, *E. sinensis* used autophagy to ensure the lipids and carbohydrates were rapidly utilized, with lipid consumption predominant. However, under long-term starvation, the stored energy in the hepatopancreas was depleted, and *E. sinensis* further selected to consume hepatopancreatic cells and maintain energy metabolism through apoptosis. In addition, cell proliferation was blocked to reduce unnecessary energy consumption (Figure 9).

## Figures and Tables

**Table 1:** Primers of various genes detected with qPCR.

Gene	Symbol	Primers	Amplicon (bp)	*T*_m_ (°C)
*Intracellular triacylglycerol lipase*	*es_IL*	F: CCTACTGCTGGATGCTGCTCTG	197	59
		R: CGACACGGAGAAGACGCAAGAC		
*Fatty acid synthase*	*FAS*	F: TTCACCAAGCCACCGTCCTCT	113	59
		R: GCCAGAAGCAGCAACAGAGTCA		
*Acyl-CoA synthase*	*ACS*	F: CCTGTCAACTGGAATAATGGCT	121	59
		R: CACTGGGCTTGTTTCTGTCAT		
*Acetyl-CoA carboxylase*	*ACC*	F: ATCAACCGCCTCCGAACCTACG	172	58
		R: CGAACGATGCCTCCTTGGTGAT		
*Autophagy-related 5*	*ATG5*	F: ACCAGCAGGACGCAGAGATGT	134	60
		R: GTGTGAGAAGTGTGCCGTGAGG		
*Autophagy-related 7*	*ATG7*	F: ACGGCTGCTCTGGGCTTTGA	180	60
		R: CGTGCGGTCGTGTGTGGAAT		
*Microtubule-associated protein 1 light chain 3*	*LC3*	F: CTAAGGCTCGCATCGGTGAC	191	60
		R: CTCGTGGTGCTCCTGGTAGA		
*Autophagy-related 9*	*ATG9*	F: CGCCGCATCAACCTAACTCTGG	104	60
		R: CGCCCGCTGAAAGGACACAA		
*Autophagy-related 12*	*ATG12*	F: AAGAAGAAGAAGTGGGCGGTG	104	60
		R: TACACAAACAGGGAGTCGCT		
*Caspase-3*	*Casp-3*	F: GGCACGAACACCGACACCTT	168	59
		R: ATCCAGGGCACATCCAGTCTCA		
*Caspase-7*	*Casp-7*	F: CTGCCAACCCTCCTCCTGAAGA	134	59
		R: GCGAGCATCACTGTCATCTGGA		
*Caspase-8*	*Casp-8*	F: GGTGGAGTTGTCAGAGGAGGGA	120	59
		R: TGGTGGAGGGCAGCATTGAC		
*BCL2-associated X*	*Bax*	F: TGAAGCAGACCACGCCATACCT	106	58
		R: ACGGTTTCTACGGTGGGTGAGT		
*B cell lymphoma-2*	*Bcl-2*	F: GGAACCTGTGGCGTCTAAGC	162	60
		R: GTGGTGGTGGTGGTGGAGTT		
*Apoptosis-inducing factor*	*AIF*	F: ACCACCAAGGACACGCCAAAG	190	59
		R: AGAGGACGATGCCCACCACAA		
*β-Actin*	*β-Actin*	F: GGCTACACCTTCACGACCAC	184	59
		R: TACACAAACAGGGAGTCGCT		
*Glyceraldehyde-3-phosphate dehydrogenase*	*GAPDH*	F: AGGTGAAGGCAGAGGATGGAG	167	60
		R: ACCAGTGAAGTGAGCAGAGGC		

## Data Availability

The data used to support the findings of this study are included within the article, and the original data are available from the corresponding author upon request.
